# The Combination of 2D Layered Graphene Oxide and 3D Porous Cellulose Heterogeneous Membranes for Nanofluidic Osmotic Power Generation

**DOI:** 10.3390/molecules26175343

**Published:** 2021-09-02

**Authors:** Pan Jia, Xinyi Du, Ruiqi Chen, Jinming Zhou, Marco Agostini, Jinhua Sun, Linhong Xiao

**Affiliations:** 1 Hebei Key Laboratory of Inorganic Nanomaterials, College of Chemistry and Material Science, Hebei Normal University, Shijiazhuang 050024, China; jiapan@hebtu.edu.cn (P.J.); duxinyi0802@163.com (X.D.); zhoujm@mail.hebtu.edu.cn (J.Z.); 2 Materials and Manufacture, Department of Industrial and Materials Science, Chalmers University of Technology, 41296 Göteborg, Sweden; ruiqichen7710@gmail.com (R.C.); jinhua@chalmers.se (J.S.); 3 Department of Physics, Chalmers University of Technology, 41296 Göteborg, Sweden; agostini.marco@hotmail.it; 4 Department of Organismal Biology, Uppsala University, 75236 Uppsala, Sweden

**Keywords:** 2D nanofluidics, graphene oxide, reversed electrodialysis, ion transport, osmotic power, energy generation

## Abstract

Salinity gradient energy, as a type of blue energy, is a promising sustainable energy source. Its energy conversion efficiency is significantly determined by the selective membranes. Recently, nanofluidic membrane made by two-dimensional (2D) nanomaterials (e.g., graphene) with densely packed nanochannels has been considered as a high-efficient membrane in the osmotic power generation research field. Herein, the graphene oxide-cellulose acetate (GO–CA) heterogeneous membrane was assembled by combining a porous CA membrane and a layered GO membrane; the combination of 2D nanochannels and 3D porous structures make it show high surface-charge-governed property and excellent ion transport stability, resulting in an efficient osmotic power harvesting. A power density of about 0.13 W/m^2^ is achieved for the sea–river mimicking system and up to 0.55 W/m^2^ at a 500-fold salinity gradient. With different functions, the CA and GO membranes served as ion storage layer and ion selection layer, respectively. The GO–CA heterogeneous membrane open a promising avenue for fabrication of porous and layered platform for wide potential applications, such as sustainable power generation, water purification, and seawater desalination.

## 1. Introduction

Salinity gradient energy is a type of blue energy and has attracted significant attention from researchers; its rapid development relies on nanotechnology and membrane science [1,2,3]. To optimize the osmotic power generation of the sea–river system, various technologies and membrane design methods for efficient energy conversion have been developed [4,5,6]. The reversed electrodialysis (RED) approach, where an osmotic current is generated from a permselective membrane, is considered to be the most promising method for salinity gradient energy harvesting compared with the pressure-retarded osmosis (PRO) [7,8,9]. For the RED approach, only the ions with opposite charge polarity could pass through the permselective membrane, resulting in the direct conversion of salinity gradient energy into electrical energy [10,11,12]. The ionic selectivity of the permselective membrane is a fundamental factor in the RED energy harvesting, which is affected by surface charge and pore/channel size [1]. Recently, a variety of nanoporous structures are employed for RED, such as one-dimensional (1D) single nanopores [13,14,15,16,17], nanochannels [18,19], and nanotubes [20,21]. However, despite the high power density, the fabrication of these materials is not facile and poorly scalable, with high cost and complex technology, which hinders its practical applications [22,23]. Macroscopic membranes with densely packed nanofluidic channels are therefore highly desired to solve such problems.

Inspired by nacre, in which soft materials are sandwiched between hard inorganic layers, two-dimensional (2D) nanofluidic membrane, assembled by exfoliated 2D nanosheets via a simple vacuum filtration process, allows independent transport of mass and charge in both vertical and horizontal directions [24,25,26]. These membranes can be scalably fabricated and easily pre-assembled with high flux and tunable channel size, providing an ideal platform for efficient nanofluidic ion transport [22,27]. The graphene oxide (GO) nanosheet is one of the most typical 2D nano building blocks to fabricate flexible and freestanding membranes. Due to the presence of oxygen-containing functional groups between the graphene layers, the assembled film could form 2D nanochannels. In addition, those functional groups on the surface of GO allow the solvent and ions to intercalate into the channels, resulting in a varied interlayer distance [28,29,30]. For example, we found that the interlayer distance of GO increase to 8.9 Å from 6.5 Å after intercalation of one layer of acetonitrile molecules, and expand to 12.7 Å with the intercalation of another layer of solvent molecules, and further increase to 16.4 Å after the co-intercalation of ions [28]. In addition, we also demonstrated that the addition of molecules (e.g, aminobenzene) with a larger size than solvent molecules and ions results in an enlarged interlayer distance with permanent size [31,32,33,34].

The nanochannels formed by graphene layers allowed the diffusion of solvent molecules and selectively trap ions depending on the properties of the graphene surface and size of nanochannels [33]. In recent years, with the development of liquid processing of colloidal 2D materials, the GO membrane has attracted enormous attention in nanofluidic applications because of its rich surface functional groups and easy processing [22]. GO membranes can be constructed with a facile vacuum filtration process [35,36,37]. The fabricated multilayer GO membranes show exceptional scalability and stability [35], thus their potential applications in ions sieving, water desalination, energy conversion, and storage [38,39,40,41]. For example, Fang et al. demonstrate ion sieving in GO membranes via cationic control of interlayer spacing [38]. Recently developed planar heterogeneous GO membranes have realized electric-field-induced ionic sieving and are used for miniaturized water desalination [39]. On the other hand, the porous cellulose acetate (CA) membrane is one of the commonly used substrate materials in the nanofluidic field due to its high stability, flexibility, and tunable channel properties [42]. In the porous CA, the pores are connected with the formation of a 3D network, while the 2D nanochannels are formed in the GO membrane. In addition, the surface chemistry and properties of GO and CA are completely different, which could result in different ions/solvent transport properties and performance. Overall, we expected that the combination of 2D layered GO membrane with 3D porous CA membrane could show unique ion transport phenomenon.

Herein, we fabricated a GO–CA membrane by depositing GO nanosheets on the CA membranes, forming a membrane with different porous structures and surface properties on the two sides of the membrane. Since the obtained film has different structures on its two surfaces, it can be termed as GO–CA heterogeneous membrane. The porous CA membrane and layered GO membrane serve as ion storage layer and ion selection layer, respectively, facilitating the cations transport from CA to GO membrane. The unique structure formed within the GO–CA heterogeneous membrane enables it to generate power due to the salinity gradient created by the GO–CA membrane. The power density of the GO–CA energy harvesting system is about 0.13 W/m^2^ for the sea–river mimicking water system, and up to 0.55 W/m^2^ for a 500-fold salinity gradient. Combination of the porous and layered structure within one membrane paves avenues for wide potential applications, such as sustainable power generation, water purification, and seawater desalination. 

## 2. Results and Discussion

### 2.1. Fabrication and Characterization of the GO–CA Heterogeneous Membrane

**Figure 1:**
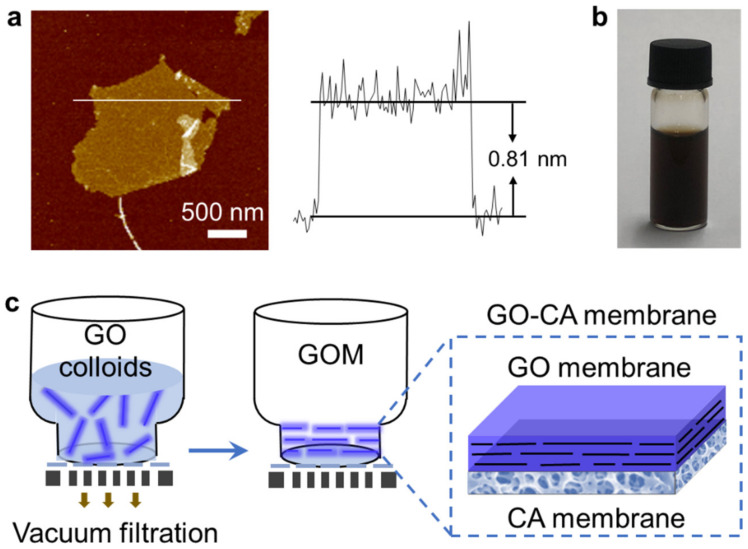
Fabrication of the GO–CA membrane: (**a**) AFM image and height profile of GO nanosheets; (**b**) photograph of the exfoliated GO dispersion (1.0 mg mL^−1^); (**c**) scheme of the vacuum filtration process for the fabrication of the GO–CA heterogeneous membrane.

The GO nanosheets were prepared from graphite powders by a modified Hummers method [43,44,45]. As shown in the atomic force microscopy (AFM), the thickness of the prepared GO nanosheet is about 0.81 nm, and its lateral size is several microns (Figure 1a). The GO nanosheets can be well dispersed in water with a brown color for months (Figure 1b). This allows us to prepare well-aligned GO film via the filtration method. The GO colloids are negatively charged in water, due to the presence of a carboxylic group on the edges of GO nanosheets. The zeta potential measurement shows that the surface charge is −43.3 mV in a neutral solution (Figure S1). The membrane composed of one layer of layered GO membrane and one layer of CA membrane is fabricated by depositing GO nanosheets on the CA membrane through vacuum filtration, termed as GO–CA heterogeneous membrane (Figure 1c). The thickness of GO film can be modulated by varying the loading of the GO dispersion. It should be noted that the low concentration of GO dispersion used for the filtration is crucial to keep all GO nanosheets being parallelly aligned on the surface of CA. Considering the large lateral size of GO used in this study, which is different from previous works, the prepared GO membrane is denser. Owing to the pores on the CA surface, the GO film stacked very well on CA film.

**Figure 2:**
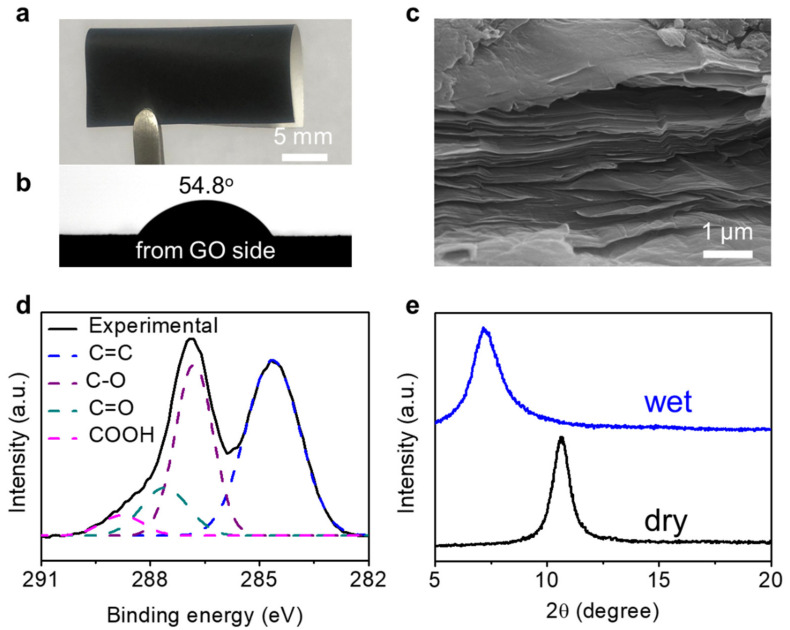
Characterization of the GO–CA membrane: (**a**) photograph of the flexible GO–CA heterogeneous membrane; (**b**) surface contact angle measurement on the GO membrane (54.8 °); (**c**) SEM observation on the cross section of the GO membrane shows a lamellar structure; (**d**) XPS spectra of the GO membrane; (**e**) XRD patterns of wet and dry GO–CA heterogeneous membrane.

The as-prepared GO–CA heterogeneous membrane is flexible (Figure 2a) and hydrophilic (the water contact angles are 54.8° and 59.2° for the GO and CA membranes, Figure 2b and Figure S2, respectively). The cross-sectional scanning electron microscope (SEM) of the GO membrane shows a typical lamellar structure with a thickness of about 3 μm (Figure 2c). The SEM observation on the surface and cross section of the CA membrane shows a porous structure with a pore size of hundred nanometers (Figure S3). The open pores within the CA form a 3D cross-linked porous network which allowed the fast penetration of solvent molecules and solvated ions. The unique structure features of the GO–CA heterogeneous membrane make it a good platform for fast ion transport in osmotic energy conversion systems. XPS results indicate that the GO contains C–OH, C=O, and COOH groups, with a C/O ratio of about 1.6 (Figure 2d and Figure S4). The presence of those oxygen-containing functional groups makes the GO show a hydrophilic surface, and they also play an important role in solvent diffusion and charge transport (see below discussion). X-ray diffraction (XRD) characterization on dry and wet GO–CA heterogeneous membranes show diffraction peaks at 10.6° and 7.2°, indicative of interlayer spacing of 0.83 and 1.23 nm, respectively (Figure 2e). The larger interlayer distance of the wet GO–CA heterogeneous membrane than the dry membrane is due to the intercalation of water molecules between the GO layers. The intercalated water molecules form hydrogen bonds with the oxygen-containing functional groups on the GO surface. Taking into account the thickness of the GO nanosheet (~0.34 nm), the effective height of the hydrated nanochannel is about 0.89 nm, which is large enough to allow most hydrated ions to intercalate and diffuse, without any barrier to transport from one side to another side [46]. The GO–CA heterogeneous membrane can keep stable in water and ionic solution for at least 8 days (Figure S5).

### 2.2. Charge-Governed Ion Transport through GO–CA Heterogeneous Membrane

**Figure 3:**
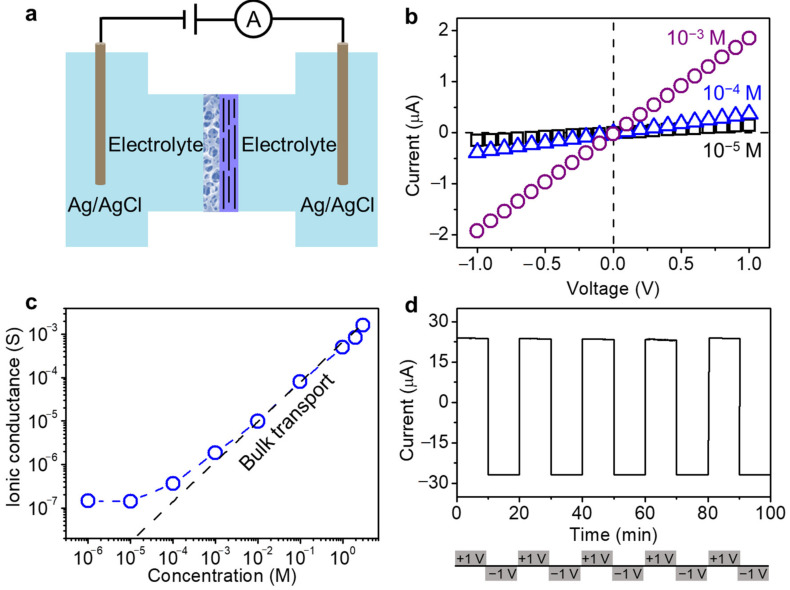
Charge-governed ion transport through the GO–CA heterogeneous membrane: (**a**) ionic transport measurement through GOM; (**b**) *I–V* characteristics of the GO–CA heterogeneous membrane for varying KCl concentrations; (**c**) ionic conductance of GO–CA heterogeneous membrane as a function of KCl concentration, which deviates from bulk value (dashed line) at 10^−2^ M, indicating the charge-governed ion transport; (**d**) time-dependent current of GO–CA heterogeneous membrane recorded in 0.1 M KCl with an external bias alternating between +1 and −1 V (20 min per cycle), showing excellent stability.

The ion transport across the GO–CA membrane was investigated by mounting a piece of membrane between a two-compartment electrochemical cell (Figure 3a). A pair of Ag/AgCl electrodes placed in two reservoirs was employed to record the ionic current. With equivalent electrolyte solution filled in the two reservoirs, Figure 3b shows the representative current–voltage (*I–V*) curves at different KCl concentrations with linear properties, indicating the transport of K^+^ from one reservoir to the other one under the applied potential. The ion transport rate can be quantified by the ionic conductance, corresponding to the slope of obtained *I–V* curve.

After calculation, the ionic conductance of GO–CA heterogeneous membrane was plotted in a wide range of electrolyte concentrations from 1 M down to 10^−6^ M, exhibiting two distinct characteristic behaviors (Figure 3c). A transition point can be observed in the plot at electrolyte concentration about 10^−2^ M. When the electrolyte concentration is higher than 10^−2^ M, the ionic conductance increases linearly with the electrolyte concentration, following the bulk transport of ions. While at a lower concentration, the ionic conductance deviates from the bulk behavior. This is because the small channels formed by charged GO surface have a significant influence on the transport of ions, resulting in the increased thickness of the Debye length, suggesting the strong surface-charge-governed ion transport property [47,48]. This is important for power generation since the ions’ transport will not be governed by the concentration but the surface charges.

To verify the stability of ion transport through the GO–CA heterogeneous membrane, the current–time (*I–t*) curve was recorded with an external bias alternated between +1 V and −1 V. Each cycle was sustained for 20 min in 0.1 M KCl solution and repeated for 5 cycles. Figure 3d shows that the positive and negative current stayed at the same level, and no decay was observed, indicating the extraordinary stability of GO–CA heterogeneous membrane for ions transport. Additionally, the membrane keeps intact after the test for 12 h (Figure S6). This can be attributed to the large lateral size of GO nanosheets used for the preparation of transport film and the mechanical support from the porous CA film. In addition, the 3D porous network in CA film acting as small reservoirs allows the fast diffusion of ions. 

### 2.3. Salinity Gradient Power Generation

**Figure 4:**
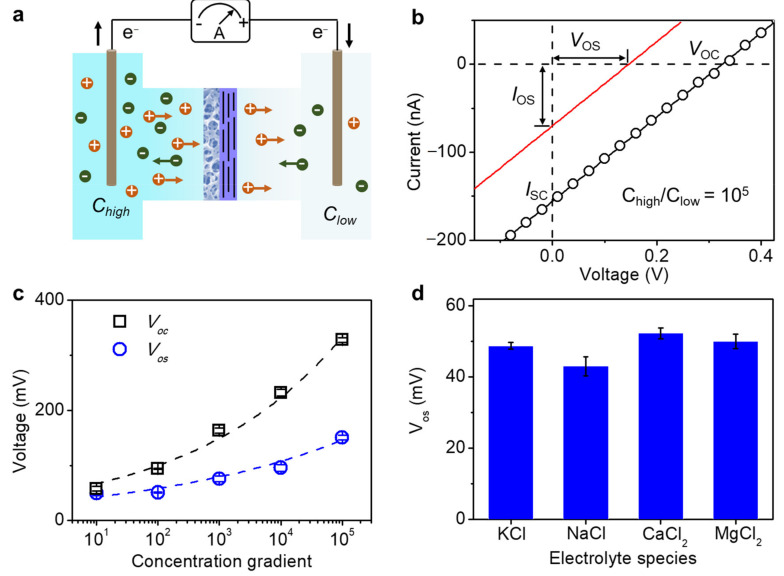
Salinity gradient power generation: (**a**) scheme of the device; (**b**) current–voltage characTable 105. across the membrane. The contribution from the redox reaction on the electrodes is subtracted from the measured current (black line), resulting in the red line, which represents the pure electroosmotic contribution. *V*_oc_ and *I*_sc_ are the open-circuit voltage and short-circuit current, respectively; *V*_os_ and *I*_os_ are osmotic voltage and current; (**c**) the open-circuit voltage and the osmotic potential of GO membrane as a function of concentration gradients (the low side is 1 μM); (**d**) the osmotic potential of GO–CA heterogeneous membrane measured in different electrolytes under a 100-fold concentration gradient.

The high surface-charge-governed ion transport properties and excellent stability of the GO–CA paved the way for salinity gradient power generation. The energy conversion performance was carried out by scanning *I–V* curves with a concentration gradient across the membrane (Figure 4a); for example, the concentration of ions in the left reservoirs is higher than that in the right reservoir. The ions would be self-driven to diffuse from the high-concentration side (*C_high_*) to the low-concentration side (*C_low_*). The recorded ionic current increases with the concentration gradient. The surfaces of channels formed by GO are negatively charged, resulting in excellent cation selectively. Almost only the cations could pass the membrane, leading to the ionic flow [49]. With the help of electrochemical redox reactions on the electrodes, the salinity gradient could be converted into electricity. Typically, with a concentration gradient of 10^5^-fold (1 μM/0.1 M KCl), the short-circuit current (*I_sc_*, −156 nA) and open-circuit voltage (*V_oc_*, 326 mV) could be obtained from the intercepts on the current and voltage axes, respectively (Figure 4b). It should be noted that the *V_oc_* is composed of the pure osmotic potential (*V_os_*) and the redox potential (*V_redox_*) caused by the reactions on electrodes (Figure S7) [14,20,40,50]. The *V_os_* and osmotic current (*I_os_*) can be obtained by subtracting the contribution of *V_redox_* of the electrodes (Materials and Methods Section). The micrometer thick GO film, which could be stabilized by depositing on porous CA film, would also allow the fast diffusion of ions from one side to the other side, compared with the thick GO film.

To further investigate the power generation ability of the GO–CA heterogeneous membrane, the *I–V* curves under various concentration gradients were tested. *C_low_* was kept at 1 μM, while *C_high_* was elevated from 10 to 10^5^ μM. As shown in Figure 4c, both the *V_oc_* and *V_os_* increase with the concentration gradient, and the maximum values are up to 326 and 151 mV, respectively, at a concentration gradient of 10^5^. It is possible to reach high voltage under an even larger concentration gradient.

Except for the salinity concentration gradient, the influence of species of ions on the ion transport behavior was also investigated. Similar salinity gradient power generation can be found with many mono- and divalent ions; for example, K^+^, Na^+^, Ca^2+^, and Mg^2+^ (Figure 4d). As for the monovalent cations under the same concentration, the *V_os_* value obtained from NaCl is lower than that of KCl, which might be due to the increased hydration radius of the cations. Additionally, for divalent ions, the *V_os_* is higher than monovalent ions, which is attributed to the fact that the bivalent ions carry two charges, while the monovalent ions only have one charge. For the voltage obtained from CaCl_2_ is high than MgCl_2_, the reason is the same with monovalent ions.

**Figure 5:**
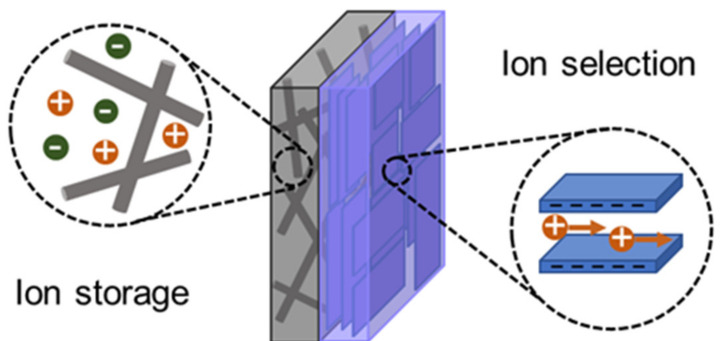
Schematic of the GO–CA membrane structure. The CA membrane and GO membrane serve as ion storage layer and ion selection layer, respectively.

In the GO–CA heterogeneous membrane system, the CA membrane and GO membrane serve for ion storage and ion selection, respectively, which benefit the fast ion transport in the reverse electrodialysis system (Figure 5). The porous CA membrane with a large pore volume could store a large number of ions, which could transfer ions to the layered GO membrane with high cation selectivity [51]. The combination of 3D porous CA film and GO film with 2D channels could allow the fast diffusion and high selectivity of ions and also promise the good stability of the film in the salt solution.

### 2.4. GO–CA Heterogeneous Membrane-Based Power Generator for Osmotic Energy Harvesting

The harvested power can be used to supply an external load resistance (*R_L_*), and the output power density can be calculated with the equation [18] as follows:

*P_max_* = *I*^2^ × *R_L_* (1)

**Figure 6:**
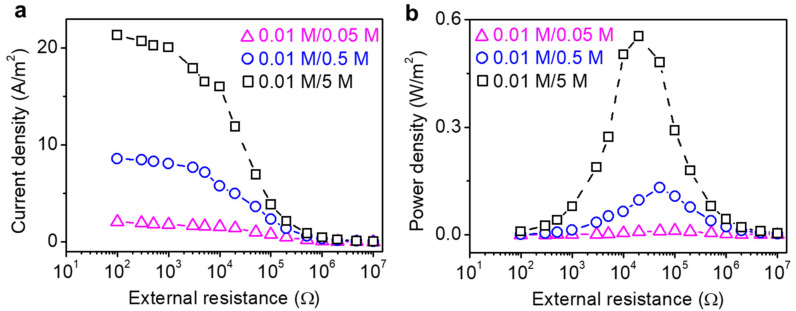
GO–CA membrane-based power generator for osmotic energy harvesting. Current density (**a**) and power density (**b**) of GO–CA heterogeneous membrane with different NaCl salinity gradients.

The output current density decreases accordingly with the increase of *R_L_*, while increases with the concentration gradient (Figure 6a). The current density obtained from a concentration gradient of 0.01 M/5 M is two times larger than that obtained from 0.01 M/0.05 M by supplying energy to a resistance of 100 Ω. The output power density achieved a maximum value at a moderate external load resistance, and the corresponding output power densities are 0.011 W m^−2^, 0.13 W m^−2^, and 0.55 W m^−2^ for the 5-fold, 50-fold, and 500-fold NaCl concentration gradient, respectively (Figure 6b). The maximum power density is achieved when the external load resistance is equal to the internal resistance [52]. For the sea–river mimicking water system (0.5 and 0.01 M NaCl), the maximum power density is 0.13 W m^−2^. Additionally, for a high salinity gradient solution, for example, 500-fold, the maximum power density reaches up to 0.55 W m^−2^ when the external resistance is about 20 KΩ. This result indicates that our GO–CA heterogeneous membrane shows a promising application for power generation even using seawater. When the membrane thickness is lower than 3.6 μm, the output power density increases with the membrane thickness, while decreases when the thickness is higher than 3.6 μm (Figure S8). The transference number and resultant output power density of the GO–CA membrane is higher than the performances of some state-of-the-art nanostructured membranes and commercial anion exchange membranes (Table S1).

## 3. Conclusions

In summary, GO–CA heterogeneous membranes were fabricated by depositing well-aligned GO nanosheet on a 3D porous CA film. The micrometer thick GO membrane with charged channels could govern the transport of ions but also allow their fast diffusion. The 3D porous CA film acting as a support to make sure the micrometer thick GO film has high mechanical stability, meanwhile the pore as reservoir supplies ions for the fast intercalation into GO film. The as-prepared GO–CA heterogeneous membrane was employed to fabricate the RED device for osmotic energy harvesting. This graphene-based nanofluidic device shows high surface-charge-governed ion transport property and excellent stability and exhibits good performance in osmotic energy conversion. A power density of about 0.13 W/m^2^ is obtained for the sea–river mimicking system and reaches 0.55 W/m^2^ under a salinity gradient of 500-fold. The porous CA membrane and layered GO membrane serve for the ion storage layer and ion selection layer, respectively. This work provides a promising avenue for the development of porous and layered hybrid membranes for nanofluidic osmotic energy harvesting.

## 4. Materials and Methods

### 4.1. Materials

Graphite powders (99.95%, metals basis) were purchased from Qingdao Xinghe graphite Co., Ltd. (Qingdao, China). GO was synthesized from the graphite powders by a modified Hummers method [43,44]. GO dispersion was further purified by dialysis and cycles of centrifugation and dispersion in water. Other chemical reagents were all analytical grade and used as received. All solutions were prepared using deionized water (18.2 MΩ cm).

### 4.2. Membrane Fabrication

GO–CA membranes were fabricated via vacuum filtration (Figure 1c). In brief, 10 mL GO dispersion (1 mg mL^−1^) was filtrated through a CA membrane (50 mm diameter, 0.2 μm nominal pore size). The prepared GOMs were dried and stabilized at 80 °C for 12 h in an oven [53].

### 4.3. Characterization

AFM images were recorded on MultiMode 8 AFM (Bruker, Bremen, Germany) in the tapping mode using a silicon cantilever for the geometry of individual GO nanosheets deposited on mica. SEM images were obtained by using a field emission SEM (Hitachi S-4800, Tokyo, Japan) with an acceleration voltage of 10 kV. Contact angle measurement was carried out on an OCA25LHT (Dataphysics, Filderstadt, Germany) instrument at room temperature. XRD patterns were collected on a Bruker D8 Focus diffractometer using an incident wavelength of 1.5418 Å (Cu Kα radiation). The zeta potential of GO colloids (0.1 mg mL^−1^) was measured with a Malvern Zetasizer NanoZS90.

### 4.4. Electrical Measurements

The ionic transport properties and the energy conversion tests were recorded by a Keithley 6487 (Cleveland, USA) semiconductor picoammeter in homemade electrochemical cells. The membrane was mounted between the two-chamber testing cell. The effective testing area was 0.2 mm^2^. A pair of Ag/AgCl compounds were used as electrodes. The conductivity of bulk electrolyte solution was measured in the same condition only without the membrane.

### 4.5. Potential Calibration

By measuring the *I–V* response, we can measure the open-circuit voltage (*V_oc_*) and the short-circuit current (*I_sc_*). The measured *V_oc_* is composed of the osmotic potential (*V_os_*) and the redox potential (*V_redox_*), which are generated by the membrane and unequal potential drop at the electrode–solution interface in the different electrolyte concentrations, respectively [54]. The *V_os_* and *I_os_* can be obtained by subtracting the *V_redox_*. The relationship is as follows:

V_os_ = V_oc_ − V_redox_

The device is the same as Figure 4, and the *I–V* curves are collected from −0.2 V to 0.2 V (step of 0.01 V) with and without the GO–CA heterogeneous membrane, respectively. The potential obtained without the membrane was regarded as *V_redox_*.

## Data Availability

The data presented in this study are available in Supplementary Material.

## Supplementary Materials

The following are available online. Figure S1: Zeta potential of GO nanosheets in neutral colloids (0.1 mg mL^−1^), Figure S2: Contact angle of CA membrane, Figure S3: SEM observation on the surface and cross section of the CA membrane show porous structure, Figure S4: XPS survey spectra of the GO membrane, implying the existence of oxygen functional groups on the surface, Figure S5: The stability of GO–CA membrane in water, KCl, and NaCl solutions, Figure S6: The morphology of GO–CA membrane before and after the test for 12 h, Figure S7: Equivalent circuit of ion transport through the GO–CA heterogeneous membrane, Figure S8: Membrane thickness dependence of the power density, Table S1: Power generation performance of GO–CA membrane compared with state-of-the-art nanostructured membranes and commercial anion exchange membranes.

## Sample Availability

Samples of the membranes and devices are available from the authors.
