# Supplementary material for: The Combination of 2D Layered Graphene Oxide and 3D Porous Cellulose Heterogeneous Membranes for Nanofluidic Osmotic Power Generation
Source: Molecules. 2021 Sep 2;26(17):5343. doi: 10.3390/molecules26175343 (PMC8434357; doi:10.3390/molecules26175343)
Supplement: Supplementary file 1 [file molecules-26-05343-s001.zip › molecules-1348700-supplementary.pdf]

Supplementary Material

2D Layered Graphene Oxide and 3D Porous Cellulose Heterogeneous Membranes as Nanofluidic Osmotic Power Generators

Pan Jia[1], Xinyi Du[1], Ruiqi Chen[2], Jinming Zhou[1], Marco Agostini[3], Jinhua Sun[2], and Linhong Xiao[4,*]

[1] Key Laboratory of Inorganic Nanomaterials of Hebei Province, College of Chemistry and Material Science, Hebei Normal University, Shijiazhuang 050024, P.R. China

[2] Materials and Manufacture, Department of Industrial and Materials Science, Chalmers University of Technology, Göteborg 41296, Sweden

[3] Department of Physics, Chalmers University of Technology, Göteborg 41296, Sweden

[4] Department of Organismal Biology, Uppsala University, Uppsala 75236, Sweden

* To whom correspondence should be addressed: linhong.xiao@ebc.uu.se

Table of Content:

1. Zeta potential of GO colloids.

2. Characterization of the CA membrane.

3. XPS of GO membrane.

4. Stabilization of GO-CA membranes in water.

5. Equivalent circuit.

6. Membrane thickness dependence of the power density.

7. Comparison with other membranes.

8. References.

1. Zeta potential of GO colloids.

We test the surface charge of GO nanosheets in neutral colloids (0.1 mg mL^{-1}). The zeta potential of GO colloids is −43.3±2.3 mV, which confirm the negatively charged property in water [1] (Figure S1).

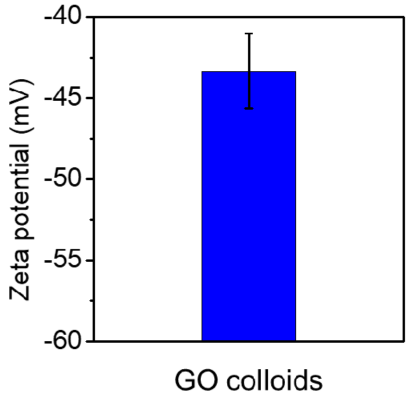

Figure S1. Zeta potential of GO nanosheets in neutral colloids (0.1 mg mL^{-1}).

2. Characterization of the CA membrane.

The properties of CA membrane were investigated. Surface contact angle measurement on CA membrane shows a hydrophilic surface (59.2º, Figure S2). SEM observation on the surface and cross section of the CA membrane show porous structure with pore size of hundred nanometers (Figure S3). The thickness of the CA membrane is about 100 μm.

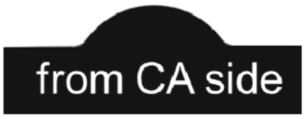

Figure S2. Contact angle of CA membrane.

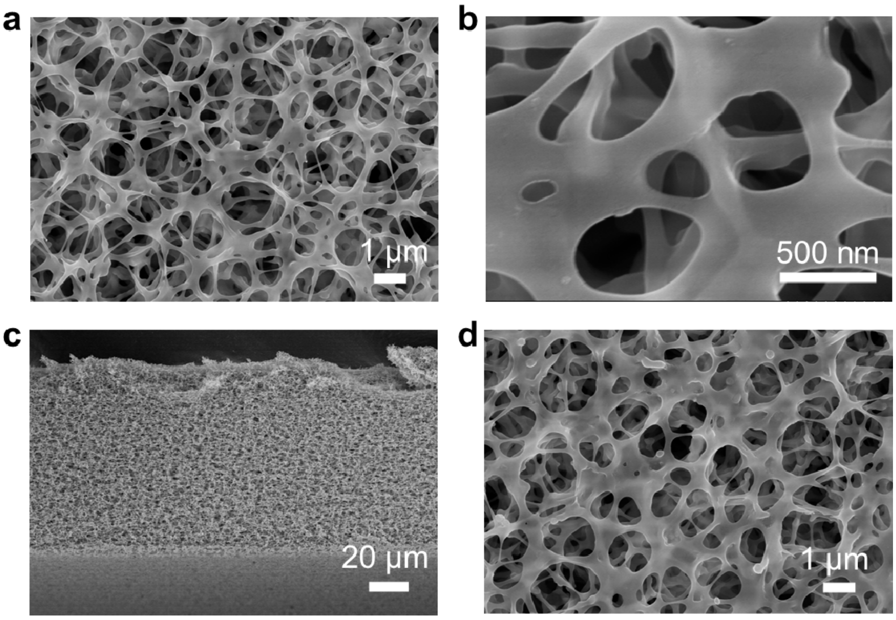

Figure S3. SEM observation on the surface (a,b) and cross section (c,d) of the CA membrane show porous structure.

3. XPS analysis of GO membrane.

To estimate the oxygen containing functional groups on GO sheets, the GO membrane was measured using X-ray photoelectron spectra (XPS). From the XPS survey spectra, the C/O ratios was calculated to be 1.6. XPS analysis of the C 1s spectra indicate that the as-prepared GO nanosheets contains four types of carbon bonds: C=C (284.7 eV), O=C−OH (288.9 eV), C−O (286.8 eV), and C=O (287.6 eV) [2, 3] (Figure S4 and Figure 2d).

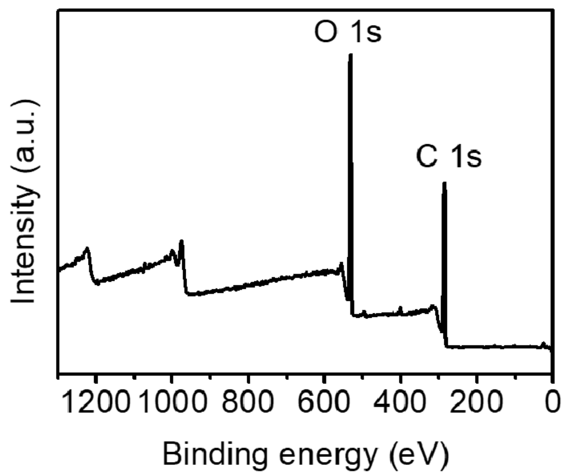

Figure S4. XPS survey spectra of the GO membrane, implying the existence of oxygen functional groups on the surface.

4. Stabilization of GO-CA membranes in water.

We tested the stability of the GO-CA membrane in water and ionic solutions. The as-prepared GO-CA membrane keeps intact in water for more than 8 days (Figure S5). Moreover, the morphology of GO-CA membrane before and after test are observed, the membrane keeps intact after test for 12h (Figure S6).

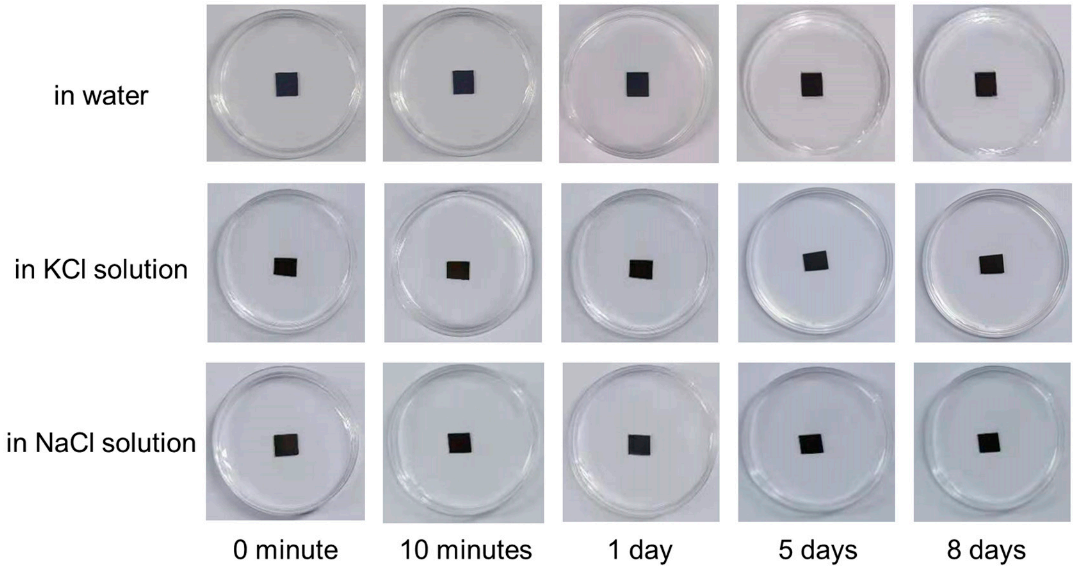

Figure S5. The stability of GO-CA membrane in water, KCl and NaCl solutions.

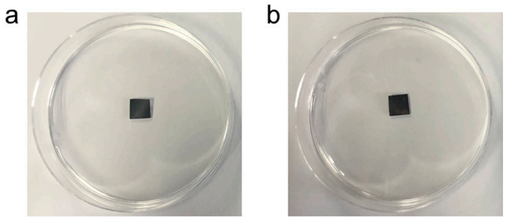

Figure S6. The morphology of GO-CA membrane (a) before and (b) after test for 12h.

5. Equivalent circuit.

The recorded open circuit voltage (V_{oc}) is composed of osmotic voltage (V_{os}) and redox voltage (V_{redox}), Figure S7 shows the equivalent circuit diagram.

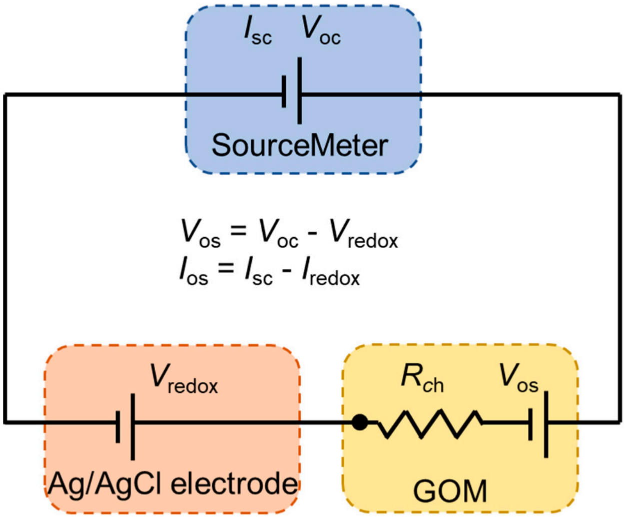

Figure S7. Equivalent circuit of ion transport through the GO-CA membrane. The V_{oc} is composed of V_{os} and V_{redox}.

6. Membrane thickness dependence of the power density.

We test the power density of GO-CA membranes with various thickness. GO-CA membranes with different thickness were prepared by varying the loading of the GO dispersion. The power density is obtained from the 0.01 M/0.5 M NaCl system with a resistance of 51 kΩ. A typical result is shown in Figure S8, when the thickness is lower than 3.6 μm, the output power density increases with the membrane thickness. While the power density decreases when the membrane thickness is higher than 3.6 μm.

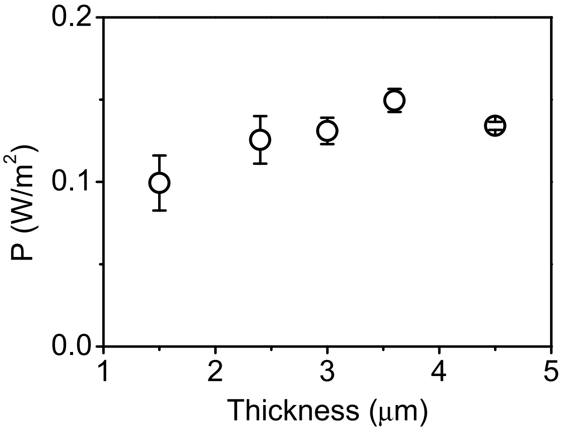

Figure S8. Membrane thickness dependence of the power density.

7. Comparison with other membranes.

We compared the GO-CA membrane with other membranes in literature and commercial anion exchange membranes (Table S1). The transference number and resultant output power density of the GO-CA membrane at high temperature is higher than the performances of some state-of-the-art nanostructured membranes and commercial anion exchange membranes.

Table S1. Power generation performance of GO-CA membrane compared with state-of-the-art nanostructured membranes and commercial anion exchange membranes.

Membrane type		Transference number	Power density (W/m^2)	Reference
This work		0.95	0.55	-
State-of-the-art nanostructured membranes	Polymeric Carbon Nitride Membranes	-	0.21	*Angew. Chem. Int. Ed.* **2018**, *57*, 10123-10126
	Ionic Diode Membrane	0.8	3.46	*J. Am. Chem. Soc.* **2014**, *136*, 12265-12272
	Nafion-filled PDMS microchannels	0.92	0.755	*Micromachines* **2016**, *7*, 205
	BCP-coated PET conical nanochannels	0.93	0.35	*J. Am. Chem. Soc.* **2015**, *137,* 14765-14772
Commercial anion exchange membranes	Ionsep (Iontech, China)	-	0.131	*J. Am. Chem. Soc.* **2015**, *137*, 14765-14772
	Neosepta (Tokuyama Corporation, Japan)	-	0.115	
	Qianqiu (Hangzhou QianQiu Industry Co., China)	-	0.106	

The value of transference number (t_+) can be obtain [4]:

$$2t_+ - 1 = \frac{E_{Diff}}{\frac{RT}{zF}\ln(\frac{\gamma_{C_H} c_H}{\gamma_{C_L} c_L})} \qquad (1)$$

where R, T, z, F, γ, C_H, and C_L represent the ideal gas constant, Kelvin temperature, charge valent, Faraday constant, activity coefficient of ions, high and low ion concentrations, respectively. For example, under a concentration gradient of 10-fold (10^{-6} M/10^{-5} M KCl), the diffusion potential was measured to be 52 mV, The value of t_+ can be calculated to be 0.95.

8. References.

1. Huang, H.; Song, Z.; Wei, N.; Shi, L.; Mao, Y.; Ying, Y.; Sun, L.; Xu, Z.; Peng, X. Ultrafast viscous water flow through nanostrand-channelled graphene oxide membranes. *Nat. Commun.* **2013,** *4*, 2979.
2. Ji, J.; Kang, Q.; Zhou, Y.; Feng, Y.; Chen, X.; Yuan, J.; Guo, W.; Wei, Y.; Jiang, L. Osmotic power generation with positively and negatively charged 2D nanofluidic membrane pairs. *Adv. Funct. Mater.* **2017,** *27*, 1603623.
3. Yang, D.; Velamakanni, A.; Bozoklu, G.; Park, S.; Stoller, M.; Piner, R. D.; Stankovich, S.; Jung, I.; Field, D. A.; Ventrice, C. A., Jr., et al. Chemical analysis of graphene oxide films after heat and chemical treatments by X-ray photoelectron and micro-raman spectroscopy. *Carbon* **2009,** *47*, 145-152.
4. Yang, J.; Hu, X.; Kong, X.; Jia, P.; Ji, D.; Quan, D.; Wang, L.; Wen, Q.; Lu, D.; Wu, J., et al. Photo-induced ultrafast active ion transport through graphene oxide membranes. *Nat. Commun.* **2019**, *10*, 1171.
